# Correlation of thermal Doppler flowmetry, brain tissue oxygen and microdialysis values in patients with severe subarachnoid hemorrhage and traumatic brain injury: a preliminary report

**DOI:** 10.1186/cc9743

**Published:** 2011-03-11

**Authors:** DC Papadopoulos, A Komnos, AS Filippidis, T Chatzopoulos, KN Fountas, G Vretzakis, K Paterakis, D Karangelis, TK Zafeiridis

**Affiliations:** 1 General Hospital of Larisa, Greece; 2 Barrow Neurological Institute, St Joseph's Hospital and Medical Center, Phoenix, AZ, USA; 3 University Hospital of Larissa, Greece

## Introduction

The purpose of this study is to investigate the relationship between continuously monitored regional cerebral blood flow (CBF), brain tissue oxygen (PbrO_2_) and microdialysis values in subarachnoid hemorrhage and traumatic brain injury patients.

## Methods

Advanced multimodal neuromonitoring including monitoring of PbrO_2 _(Licox; GMS), CBF (QFlow; Hemedex) and brain lactate, pyruvate, lactate/pyruvate ratio, glycerol and glucose values using microdialysis (CMA600; Microdialysis) was performed so far in eight patients with severe subarachnoid hemorrhage (*n *= 5) and traumatic brain injury (*n *= 3) for an average of 9.2 days. Additional recorded parameters include ICP, CPP, MABP, CVP, local brain temperature, body core temperature, PCO_2_, and blood glucose. The cerebral monitoring probes are inserted via a bolt (ICP, PbrO_2_, microdialysis) and an additional burr hole (CBF). All probes are positioned in the penumbra and location is verified by a brain CT. The study is to be conducted for an estimated total of 30 patients suffering the above pathologies.

## Results

The data so far indicate a strong correlation between CBF and PbrO_2 _values. There seems to be a link between brain glucose levels and CBF values; however, it is not as clear as regards the CBF-PbrO_2 _correlation. This may be due to the fluctuation of brain glucose because of brain ischemia, hyperemia, hypermetabolism or hypometabolism. So far we were able to establish a correlation of CBF-PbrO_2 _and lactate/pyruvate ratio only in persistently low CBF-PbrO_2 _values (CBF <12 ml/100 g/minute, PbrO2 <10 mmHg for more than 64 minutes).

## Conclusions

This is a preliminary report of a study in human patients with severe subarachnoid hemorrhage and traumatic brain injury. The results indicate correlations of varying significance between the pooled data. We hope that the outcome of our study will be able to clarify the pathophysiology of severe brain injury and guide us in the titration of therapy, as it is needed by each individual patient.
